# Fifth Eurasia Conference on Chemical Sciences

**DOI:** 10.1155/MBD.1995.244

**Published:** 1995


					FIFTH
ON
EURASIA CONFERENCE
CHEMICAL SCIENCES
The 5th Eurasia Conference on Chemical Sciences will be held in Guangzhou (Canton), P. R.
China, on 10-14 December 1996. This conference will bring scientists from the Eurasia
Supercontinent together for the further enhancement of scientific cooperation and development in
the field of chemical research.
The scientific sessions will include the following topics:
Solution coordination chemistry.
Environmental and analytical chemistry.
Bioremediation.
Rare-earth (bio)coordination chemistry.
Toxicology of metal ions.
Nanochemistry
Supramolecular chemistry and materials
Theoretical, computational and modeling chemistry
Chemistry of traditional drugs
Regulation of biosystems by metal ions and small molecules
Biopolymers and biomimetic polymers
The deadline for submission of abstracts is May 1, 1996. A 1-page camera-ready manuscript,
that will be published in the Proceedings of the Conference, will be required from participants
wishing to present a poster or an oral communication.
Registration fees for the conference will cover the payments of the participation in all the scientific
sessions, the proceedings and all printed materials of the conference, the shuttles between airport
or railway station and hotels, a welcome reception, a banquet and other scheduled social events
including one day excursion to outer suburban or inner city. Cost of registration will be US$300.
Various categories of hotel rooms will be available. Special events and tours will be organized.
Opportunities for sightseeing and tours will be provided for attendees and their spouses.
International Organizing Committee:
Jan Reedijk (Chairman),
Ivano Bertini,
Mu Shik Jhon,
Hitoshi Ohtaki,
B. Michael Rode,
A. Geoff. Sikes,
John Webb,
Salag Dhabanandana,
Youngkyu Do.
All inquiries relating to the conference should be addressed to"
Prof. Liang-Nian Ji
General Secretary EuAsC2S-1996
Biotechnology Research Center
Zhongshan( Sun Yatsen) University
Guangzhou (Canton) 510275, P. R. China
Tel: 86 20 418 54 61 or 4186 6300 7115
Fax: 86 20 418 91 73 or 86 20 418 55 51
e-mail: Leiy@ bepc2.ihep.ac.cn
244

				

